# Supplementary material for: Determining the Pharmacokinetic Properties of Two Different Doses of Meloxicam in Barred Owls (Strix varia)
Source: Animals (Basel). 2024 Oct 26;14(21):3086. doi: 10.3390/ani14213086 (PMC11545717; doi:10.3390/ani14213086)
Supplement: Supplementary file 1 [file animals-14-03086-s001.zip › animals-3265246-supplementary.pdf]

Table S1. Data for barred owls exposed to [A] 1 mg/kg and [B] 2 mg/kg meloxicam doses IM for a single treatment. Table [C] represents the barred owls treated with 1 mg/kg following the removal of birds 663 and 664 because of the unexpectedly high 4-12 hour concentrations. These results were 2.3-5.1 and 2.7-5.8; 2.5-25.9 and 2.7-27.2; and 17.9-107.7 and 14.7-88 times higher than samples for the other four owls at the 4, 6, and 12 hour sampling periods, respectively.

A

	**Dose 1 mg/kg**							
**Time**	**126**	**615**	**663**	**664**	**747**	**968**	**Mean**	**Std Dev**
0	0.15	0.04	5.74	3.40	10.23	0.16	3.29	4.11
0.5	8.23	7.59	11.12	8.75	7.86	8.94	8.75	1.27
0.75	6.35	5.54	8.48	1.05	0.46	7.95	4.97	3.44
1	5.73	5.43	7.79	0.45	5.77	6.45	5.27	2.51
2	3.23	2.66	2.76	4.69	2.23	2.84	3.07	0.86
4	0.85	0.58	2.98	3.39	1.27	0.87	1.65	1.21
6	0.30	0.11	2.85	2.99	1.12	0.30	1.28	1.32
12	0.15	0.03	3.23	2.64	0.00	0.18	1.04	1.48

B

	Dose 2 mg/kg							
**Time**	**273**	**571**	**529**	**495**	**771**	**980**	**Mean**	**Std Dev**
0	0.14	0.03	0.03	0.03	0.91	0.16	0.22	0.34
0.5	13.73	14.13	17.25	13.37	29.61	8.31	16.07	7.23
0.75	13.33	19.20	20.53	11.50	20.10	8.67	15.55	5.05
1	10.18	16.58	16.32	10.48	17.14	10.38	13.51	3.48
2	6.08	11.42	10.02	3.64	11.49	5.00	7.94	3.45
4	1.45	5.99	3.64	0.48	6.17	0.81	3.09	2.56
6	0.33	2.97	0.82	0.10	2.14	0.27	1.11	1.18
12	0.15	0.15	0.05	0.04	0.18	0.16	0.12	0.06

C

	**Revised Dose 1 mg/kg (removed outliers for 663, 664)**							
**Time**	**126**	**615**	**663**	**664**	**747**	**968**	**Mean**	**Std Dev**
0	0.15	0.04	5.74	3.40	10.23	0.16	3.29	4.11
0.5	8.23	7.59	11.12	8.75	7.86	8.94	8.75	1.27
0.75	6.35	5.54	8.48	1.05	0.46	7.95	4.97	3.44
1	5.73	5.43	7.79	0.45	5.77	6.45	5.27	2.51
2	3.23	2.66	2.76	4.69	2.23	2.84	3.07	0.86
4	0.85	0.58			1.27	0.87	0.89	0.28
6	0.30	0.11			1.12	0.30	0.46	0.45
12	0.15	0.03			0.00	0.18	0.09	0.09
